# Assessment of dermal sensitization by nickel salts in a novel humanized TLR-4 mouse model

**DOI:** 10.1080/1547691X.2024.2414979

**Published:** 2024-12-04

**Authors:** K. A. Roach, S. E. Anderson, C. Waggy, J. Aldinger, A. B. Stefaniak, J. R. Roberts

**Affiliations:** a Allergy and Clinical Immunology Branch (ACIB), National Institute of Occupational Safety and Health (NIOSH), Morgantown, WV, USA; b Office of the Director, NIOSH, Morgantown, WV, USA; c Respiratory Health Division, NIOSH, Morgantown, WV, USA

**Keywords:** Nickel, toll-like receptor-4, local lymph node assay, hypersensitivity, allergy model, sensitization

## Abstract

The fundamental goal of this study was to determine the potential utility of a novel humanized Toll-like receptor-4 (hTLR-4) mouse model for future *in vivo* studies of nickel allergy. First, mice of both sexes and hTLR-4 expression profiles were incorporated into a Local Lymph Node Assay (LLNA) to assess skin sensitization. Next, a set of hTLR-4 hTLR-4-positive mice (female and male groups) was similarly exposed to vehicle control (VC) or 10% NiSO_4_ on Days 1, 2, and 3. Mice were euthanized on Day 10, lymph node (LN) cellularity was assessed, LN and spleen cells were phenotyped, and serum was collected to quantify circulating cytokine and IgE levels. In the LLNA, hTLR-4-positive mice of both sexes exhibited enhanced responsivity to nickel. NiSO_4_ (10%) had a stimulation index (SI) of 3.7 (females) and 3.8 (males) in hTLR-4-positive animals, and an SI of 0.5 (females) and 0.8 (males) in hTLR-4 hTLR-4-negative mice. In the 10d study, hTLR-4-positive mice exposed to 10% NiSO_4_ exhibited increased LN cellularity (6.0× increase in females, 3.2× in males) and significantly higher concentrations of circulating IgE (4.1× increase in females, 3.4× in males). Significant increases in serum interferon (IFN)-γ, interleukin (IL)-4, and IL-5 levels were seen in female mice, while altered concentrations of IL-4 and IL-10 were detected in male mice. The results of this study ultimately demonstrate that murine expression of hTLR-4 confers enhanced susceptibility to dermal sensitization by nickel, and consequently, the hTLR-4 mouse model represents a viable approach for future studies of nickel allergy *in vivo*.

## Introduction

Allergic disease is widely recognized as one of the most prominent public health concerns of the modern era. Currently, it is estimated that up to 30% of the world’s population is afflicted with some form of allergic disease—the most prevalent manifestations of which include contact allergy, asthma, and rhinosinusitis, along with hypersensitivity responses caused by foods, drugs, and insect stings (Sánchez-Borges et al. 2018). These and other allergic disorders have been continually increasing in prevalence over the past several decades in many countries (Asher et al. 2006; Pawankar et al. 2013; Prescott et al. 2013). A general increase in the severity of allergic reactions has also been observed in recent years, and it has been suggested that more complex pathogenic mechanisms are becoming responsible for many hypersensitivity reactions. Moreover, the age of onset for allergic conditions has been gradually decreasing over time as well, implicating an increasing number of children at risk for these diseases (Serebrisky and Wiznia 2019). Although allergic diseases represent a significant health concern for the general public, allergies also present a prominent concern in the workplace. An estimated 11 million workers in the United States alone are at risk for exposure to potential sensitizers in their workplace and occupational exposures have been implicated in as many as 25% of all cases of allergy (Anderson et al. 2017). Overall, the expansive burden of disease associated with allergy is evidenced by significant rises in global morbidity and mortality, lost wages and compromised productivity in the workplace, direct and indirect medical costs, and profound reductions in quality of life for those afflicted with various allergic conditions (Lamb et al. 2006; Yaghoubi et al. 2019; Dierick et al. 2020).

Nickel is one of the most common contact allergens worldwide, causing allergic contact dermatitis (ACD) in an estimated 20% of the general population (Tramontana et al. 2020; Moon et al. 2021). Interestingly, unlike many other allergens, nickel allergy exhibits a widespread geographic distribution, affecting both industrialized and developing nations equally (Ahlström et al. 2019). Allergic sensitivity to nickel is also consistently observed in both male and female populations and is generally the leading cause of allergy in all age groups ranging from newborns and children to adults and the elderly (Wöhrl et al. 2003; Jacob et al. 2015; Warshaw et al. 2019). Although ACD constitutes the most prevalent manifestation of nickel allergy, the metal is also known to cause various other hypersensitivity responses (Roach and Roberts 2022). Nickel can also cause contact urticaria, ocular hypersensitivity responses, variants of oral mucosal allergy, and granulomatous allergic reactions following dermal contact (Estlander et al. 1993; Ditrichova et al. 2007). Inhalation exposure to nickel has been shown to cause hyper-sensitivity laryngitis, airborne ACD, asthma, and hypersensitivity pneumonitis in susceptible workers (Malo et al. 1985; Kusaka et al. 1991; Candura et al. 2001; Verna et al. 2002; Brera and Nicolini 2005; Buyukozturk et al. 2013; Franzen et al. 2017). Ingestion of nickel can also lead to contact allergic gastritis and allergic esophagitis in sensitized patients (Pföhler et al. 2016; Nucera et al. 2019). Finally, systemic exposures to nickel, which result primarily from the implantation of medical and dental devices and their subsequent ion release, can cause various widespread and systemic hypersensitivity responses ranging from anaphylaxis and Kounis Syndrome to systemic ACD and chronic urticaria syndrome (Abeck et al. 1993; Almpanis et al. 2010).

Despite the prevalence of nickel allergy in the general population and its implications for occupational health and safety, many of the immunological mechanisms involved in the development of nickel hypersensitivity and the subsequent elicitation of allergic responses remain unclear. The existence of this knowledge gap is, at least partially, reflective of historical challenges in inducing nickel allergy in laboratory rodents (Kimber et al. 2011). Although nickel has been repeatedly incorporated into standard immunotoxicity assays since the late 1980s, including the Local Lymph Node Assay (LLNA) (Cochrane et al. 2015), the metal consistently produces false negative results in assays of nickel sensitization (Yoshiaki, et al. 1992; Basketter et al. 1999). Moreover, the successful incorporation of nickel into various *in vivo* allergy models has required the use of adjuvants to elicit immunological responses representative of nickel allergy in humans (Johansen et al. 2010; Schmidt et al. 2011; Eguchi et al. 2013).

In recent years, a novel transgenic mouse model expressing a humanized (h) Toll-like receptor (TLR)-4 protein was developed by Hajjar et al. to study bacterial lipopolysaccharide (LPS) recognition *in vivo* (Hajjar et al. 2012). In 2010, the mouse model was also utilized by Schmidt et al. (2010) to study nickel allergy. That group was able to demonstrate, using histopathological techniques, that expression of hTLR-4 by mice confers increased susceptibility to dermal sensitization following intradermal injection of nickel salts. Ultimately, it was discovered that seemingly minor, species-specific differences in TLR-4 structure are responsible for the discrepancies in susceptibility to nickel sensitization between humans and rodents.

Consequently, the fundamental goal of this study was to assess the potential utility of the hTLR-4 mouse model for use in future *in vivo* studies of nickel allergy. Accordingly, hTLR-4-positive and negative mice of both sexes were first incorporated into an LLNA to evaluate dermal sensitization following topical exposure to soluble nickel salts. The ensuing immune responses were then characterized further by incorporating female and male hTLR-4-positive mice into a non-radioactive endpoint-based assay. Utilizing the same exposure scheme as in the LLNA, mice were euthanized following exposure and the auricular lymph nodes, spleen, and blood were collected to assess various immunological parameters associated with allergy and ACD.

## Materials and methods

### Chemicals and test materials

Nickel sulfate hexahydrate (NiSO_4_, N72–3) in powder form was purchased from ThermoFisher Scientific (Fair Lawn, NJ). Dosing solutions were prepared in mineral oil (ThermoFisher) as a delivery vehicle.

### Humanized TLR-4 mouse model

All mice used in this study were generated from an in-house breeding colony. The transgenic mouse model was originally developed in 2010 by Hajjar et al. for use in bacterial infectivity models (Hajjar et al. 2012). A humanized TLR-4 (hTLR-4) strain was established on a C57BL/6 background and is characterized by expression of human sequence-specific TLR-4 proteins by innate immune cells. The strain was preserved by Jackson Laboratory (B6.Cg-Tg(LY96)7020Haj Tg(TLR4)5271Haj/J; stock no: 031051). Female mice bearing heterozygous expression of the humanized *TLR4* gene were purchased from this colony, along with wild-type C57BL/6 males (Jackson Laboratory, Bar Harbor, ME), to as the primary breeding pairs for the in-house hTLR-4 mouse colony.

Mice were mated at 6–10 wk-of-age using standard procedures described in an existing breeding protocol approved for use in the National Institute for Occupational Safety and Health (NIOSH) Animal Facility (17–009). Several mating cycles were required to obtain an adequate number of age-matched animals for use in the studies. Pups were weaned at 5 wk postnatal for all litters. At this time point, tail biopsies were taken from each pup and sent for genotypic analysis to Transnetyx, Inc. (Cordova, TN) to determine hTLR-4 status. To maximize colony output, mice of both sexes and genotypes were incorporated into the allergy studies.

Mice were housed 4 per cage (maximum) in polycarbonate ventilated cages with HEPA-filtered air, according to genotype and sex in the NIOSH Animal Facility until 10 wk-of-age. Mice were provided food (Harlan Teklad Rodent Diet 7913) and water *ad libitum* in a controlled humidity (40–60%)/temperature (18–24 °C) environment with a 12-h light/dark cycle. All procedures in the subse-quent studies comply with the ethical standards set forth by Animal Welfare Act and the Office of Laboratory Animal Welfare (OLAW). The studies were approved by the CDC-Morgantown Institutional Animal Care and Use Committee in accordance with approved animal protocols (13-SA-M-022, 18–001).

### Local lymph node assay (LLNA)

**Figure 1.**
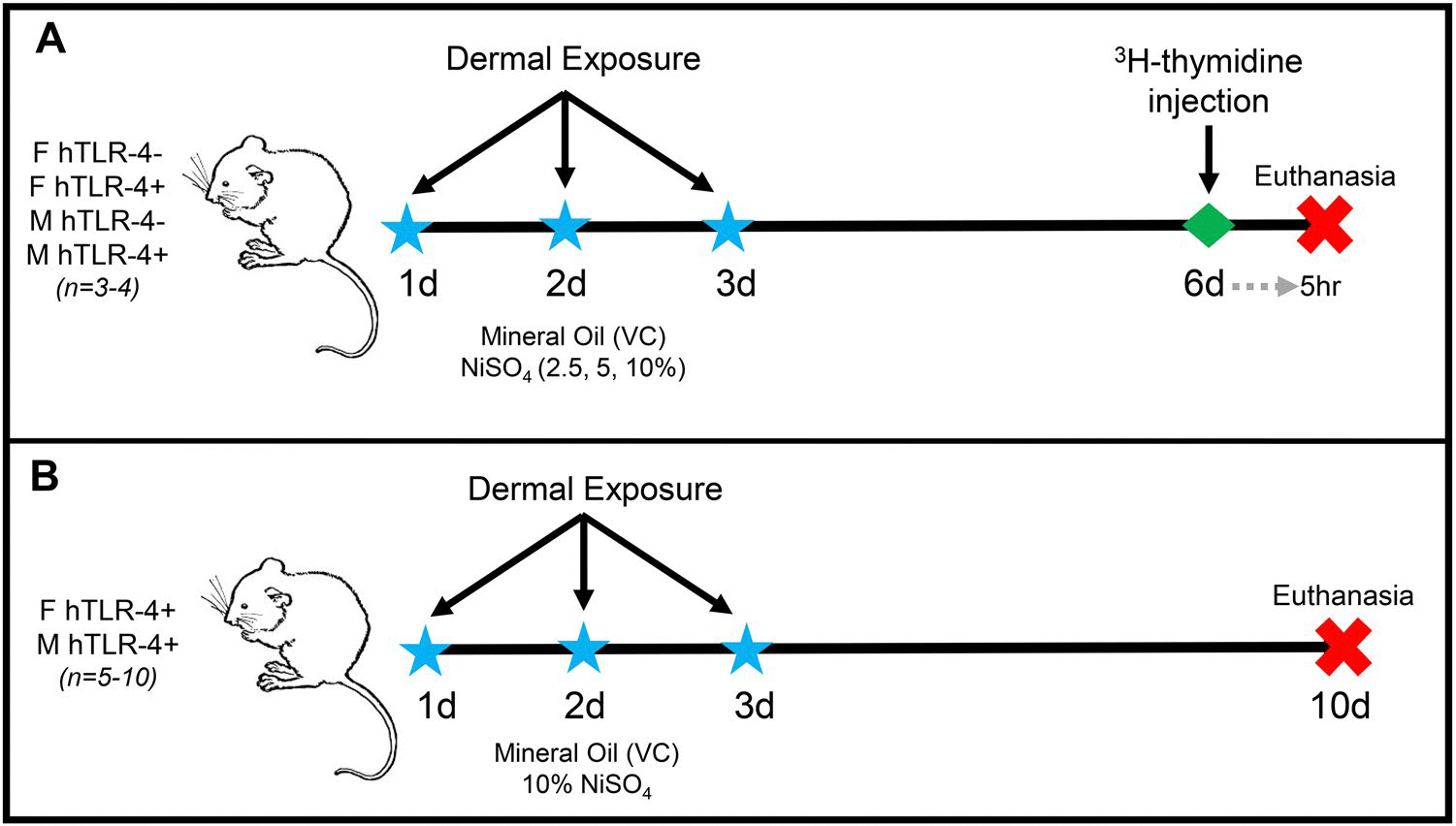
A summary of the study designs for the Local Lymph Node Assay and 10-day study are shown. (A) For the LLNA, hTLR-4-positive and -negative female (F) and male (M) mice (*n* = 3–4) were topically exposed to mineral oil (vehicle control, VC) or NiSO_4_ (2.5, 5, or 10% w/v) on days 1–3. Mice were rested for two days, then injected intravenously with tritiated-thymidine on day 6, and euthanized by CO_2_ asphyxiation 5 h later. Auricular lymph nodes were harvested from each animal, processed, and radio-isotope incorporation was assessed. (B) For the 10-d study, male and female hTLR-4-positive mice (*n* = 6–10) were topically exposed to mineral oil (vehicle control, VC) or 10% NiSO_4_ for three consecutive days, similar to the exposure scheme used in the LLNA study. Mice were rested until day 10, then euthanized by intraperitoneal injection of sodium pentobarbital euthanasia solution. The auricular lymph nodes (left and right) were harvested, total cellularity was assessed, and phenotypic analysis was performed on lymph node cell subsets. The spleen was isolated, weighed, and phenotypic analysis was performed on spleen cell subpopulations. Whole blood was collected to perform differential analyses on circulating leukocyte subsets and serum was isolated to measure circulating total IgE and cytokine concentrations.

The LLNA study was performed in accordance with established protocols and minor modifications previously described by Anderson et al. (2007). Three dosing solutions containing NiSO_4_ were prepared at concentrations of 2.5, 5.0, and 10% (w/v) in mineral oil. As shown in Figure 1A, mice (*n* = 3–4/group) were exposed topically to vehicle control (mineral oil) or NiSO_4_ on the dorsal sides of both ears (25 μl dosing solution/ear) for three consecutive days (1, 2, and 3 d). Following two days of rest (i.e., on 6 d), mice were injected intravenously *via* the lateral tail vein with 20 μCi tritiated-thymidine (specific activity 2 Ci/mmol; Dupont NEN, Boston, MA). Five hours after the thymidine injection, mice were euthanized *via* CO_2_ asphyxiation. The left and right auricular lymph nodes were excised from each mouse and processed between frosted microscope slides to yield a single cell suspension in phosphate-buffered saline (PBS). Cells were washed, re-suspended in 5% trichloroacetic acid (TCA), incubated overnight at 4 °C, then analyzed using a Packard Tri-Carb 2500TR liquid scintillation counter. Stimulation indices (SI) were then calculated by dividing the mean disintegrations per minute (DPM) for each test group by the mean DPM for the corresponding vehicle control group. Dermal sensitization in the LLNA is defined by an SI value of > 3.

### Assessment of additional immune parameters following dermal exposure to nickel

To better characterize the immune responses associated with nickel sensitization in the hTLR-4 mouse model, another study was performed without radioactivity-based endpoints. As shown in Figure 1B, hTLR-4-positive female and male mice were incorporated into the study (*n* = 5–10/group). Mice were exposed to either vehicle control or 10% NiSO_4_ using identical procedures as those employed for dosing in the LLNA study. After three consecutive days of exposure (1, 2, and 3 d), mice were rested for six days and euthanized on 10 d by CO_2_ asphyxiation. Directly following euthanasia, the body cavity was opened and blood was drawn from the abdominal aorta. For each animal, 100 μl whole blood was dispensed into EDTA-coated tubes and placed on a roller until analyses were performed. The remaining fraction was collected into a serum separator tube. The auricular lymph nodes (right and left) were collected from each animal and placed in sterile PBS until processing. The spleen was harvested from each animal, weighed, and placed in sterile PBS until processing.

### Phenotypic analysis of lymphoid tissues

Following harvest, lymph nodes and spleens were processed between frosted microscope slides to yield single cell suspensions in sterile PBS. The total number of lymph node cells was evaluated for each animal using a Multisizer II (Coulter Electronics; Hialeah, FL). Phenotypic analysis was then performed on lymph node and spleen cells by flow cytometry.

For each sample, ≈ 500,000 cells were plated and suspended in staining buffer (PBS with 1% bovine serum albumin and 0.1% sodium azide) containing F_c_ receptor blocking anti-mouse CD16/32 antibody (BD Biosciences, San Diego, CA). Cells were incubated for 5 min, washed, and then re-suspended in staining buffer containing fluorochrome-conjugated antibodies. Lymph node and spleen cells were stained with a panel of fluorochrome-labeled antibodies for cell surface markers that allowed for differentiation of lymphocyte subsets. Specifically, CD2-BV605, CD3-APC, CD4-FITC, CD8-PE, CD44-APC-R700, CD45-PerCP, CD45R(B220)-PE-Cy7, and CD86-BV421 were used to discriminate between populations of CD4^+^ T-lymphocytes, CD8^+^ T-lymphocytes, B-lymphocytes, and natural killer (NK) cells, as well as to determine the corresponding activation state. Cells were incubated for 30 min, washed, and fixed in 100 μl Cytofix Buffer (BD Biosciences).

Compensation controls were prepared using corresponding cell types stained with a single fluorophore. For each sample, 100,000 events were recorded on an LSR II flow cytometer (BD Biosciences). In all analyses, doublet exclusion was first performed by gating on SSC-A x SSC-H. Populations were then gated using FSC-A x SSC-A parameters prior to subsequent analysis. All data analysis was performed using FlowJo Software (v.7.6.5, TreeStar Inc., Ashland, OR).

### Circulating leukocyte cellular differentials

The 100-μl aliquot of whole blood retained from each animal was used for differential analysis of circulating leukocyte populations using an IDEXX ProCyte Dx Hematology Analyzer (IDEXX Laboratories; Westbrook, ME). Total leukocyte number was determined for each sample, and leukocyte subsets were differentiated to yield absolute number of lymphocytes, monocytes, neutrophils, eosinophils, and basophils.

### Serum protein analysis

The remaining volume of whole blood collected from each animal was aliquoted into a serum separation tube and centrifuged for 10 min at 1100 g to yield serum and stored at −20 °C until analysis. To quantify circulating total immunoglobulin (Ig)-E levels, serum was diluted 1:10 and assessed by enzyme-linked immunosorbent assay (ELISA) using the Mouse IgE ELISA kit (Innovative Research; Novi, MI) according to manufacturer instructions. Upon completion of the assay, the samples were allowed to develop for 30 min and absorbance was determined at 450 nm in a Spectramax Vmax plate reader (Molecular Devices, San Jose, CA). The concen-tration of IgE in each sample was interpolated from a standard curve using multipoint analysis.

Concentrations of various cytokines were also assessed in the serum of mice. Cytokines were quantified using a Milliplex MAP Kit magnetic bead panel (EMD Millipore Corporation, Billerica, MA) and analyzed on a Luminex 200 system (Luminex Corporation, Austin, TX). The specific analytes of interest included a panel of T-helper (T_H_) type 1-associated cytokines (IL-2, IL-12, IFNγ) and several major T_H_2-related cytokines (IL-4, IL-5, IL-13). Levels of the pleiotropic cytokines IL-6 and IL-10 were also assessed with the kit.

### Statistical analyses

Statistical analyses for all assays were conducted with Prism software (v.7, GraphPad, San Diego, CA). Results from all studies are expressed as means ± SE and considered statistically significant at *p* < 0.05. Treatment groups were compared by one-way analysis of variance (ANOVA) followed by a Tukey’s *post-hoc* test.

## Results

### LLNA study

Mice of both sexes and genotypes were incorporated into an LLNA to assess dermal sensitization by NiSO_4_ (Figure 1A). Following exposure and tritiated-thymidine injection, the auricular lymph nodes were harvested on 6 d and radioisotope uptake was assessed—a parameter reflective of lymphocyte proliferation in response to exposure. In accordance with the standardized LLNA protocol, *a* ≥ 3-fold increase in radioactivity is indicative of dermal sensitization.

**Figure 2.**
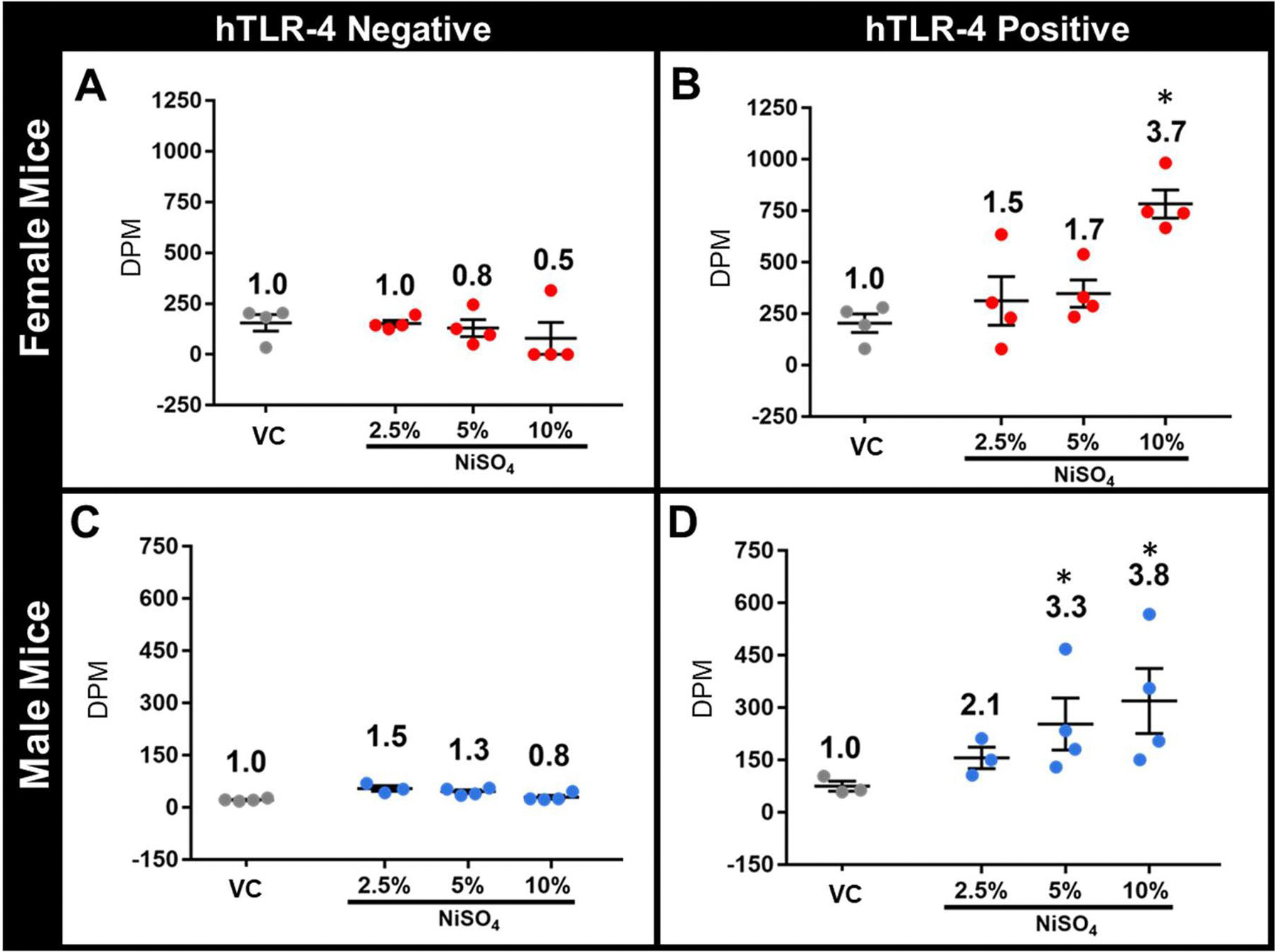
Results from the LLNA study using female (A, B) and male (C, D) hTLR-4-negative (A, C) and hTLR-4-positive (B, D) mice. Lymph node radioisotope incorporation [expressed as disintegrations per minute (DPM)] is shown for each animal. Stimulation index (SI) was calculated for each group (value listed above corresponding data points) by dividing the group average DPM by that of the respective control group. In the LLNA, an SI of ≥ 3 was considered indicative of allergic sensitization. *n* = 4, *p* < 0.05, *statistically different from same-sex control group and SI of ≥ 3 (dermal sensitization).

The results of the LLNA are shown in Figure 2. In hTLR-4-negative mice (Figure 2A, B), no discernable alterations in lymphocyte proliferation were observed after nickel exposure, irrespective of treatment group or sex. Comparatively, in hTLR-4-positive mice, notable increases in immunological responsivity to NiSO_4_ were evident among both sexes of mice, and dose-responsive increases in lymph node cellularity were observed (Figure 2B, D). In female and male mice, the maximum dose of 10% led to SI values ≥ 3 (3.7 and 3.8, respectively), indicating sensitization of these animals. In male hTLR-4-positive mice, exposure to 5% NiSO_4_ also led to an SI ≥ 3 (3.3).

### 10-Day study

The use of radioactivity-based endpoints in the LLNA fundamentally limits the potential for acquisition of additional immunological endpoints from the same group of mice. Conse-quently, another study was performed without radioactive endpoints to evaluate other indices of the immune response following dermal exposure to nickel. In this study, mice were exposed to vehicle control or 10% NiSO_4_ for three consecutive days (1 d, 2 d, 3 d), similarly to the mice of the LLNA study. Mice were then rested for 6 d and euthanized on 10 d; the auricular lymph nodes, spleen, and blood were then collected for analysis.

### Lymph node cellular analysis

**Figure 3.**
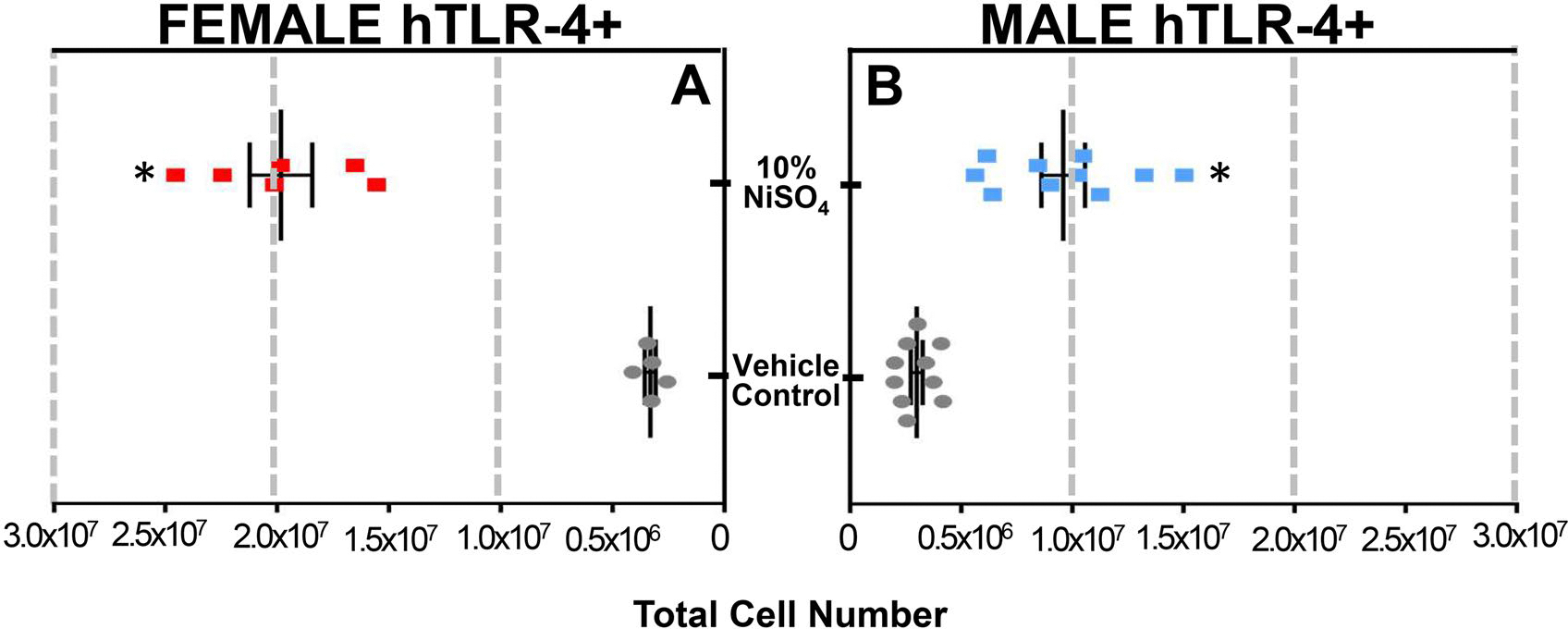
Auricular lymph node cellularity in female (A) and male (B) hTLR-4-positive mice at 10-d timepoint. In left panel (a), total cell number is shown for female mice exposed to vehicle (gray dots) or 10% NiSO_4_ (red squares). In right panel (B), total cell number is shown for male mice exposed to vehicle (gray dots) or 10% NiSO_4_ (blue squares). *n* = 6–10, *p* < 0.05, *statistically different from same-sex control group.

Significant increases in lymph node cellularity were evident in both sexes of mice at 10 d, but female mice exhibited a greater maximal response compared to males (Figure 3). In female hTLR-4-positive mice, the lymph nodes were comprised of 3.32 × 10^6^ and 1.98 × 10^7^ total cells at 10-d in VC-exposed and NiSO_4_-exposed mice, respectively. In male mice, total cell number for the VC group was 2.99 × 10^6^ and 9.59 × 10^6^ in NiSO_4_-exposed animals.

Lymph node cells were then stained for extracellular markers and analyzed by flow cytometry to differentiate immune cell subsets comprising the nodes (Table 1). Despite significant increases in total cell number among both sexes of mice exposed to NiSO_4_, only female hTLR-4-positive mice exhibited significant alterations in lymphocyte sub-population proportionality at 10 d. A significant decrease in the percentage of CD4^+^ T-cells (44.5 vs. 51.2%) comprising the nodes was observed in these mice, in addition to a significant increase in the percentage of CD8^+^ T-cells (25.1 vs. 20.1%) within the nodes.

Flow cytometric analysis was also used to determine activation status of T- and B-cell sub-popu-lations in the lymph nodes of hTLR-4-positive mice at 10 d. Consistent with the significant increase in proportionality of CD8^+^ T-cells comprising the nodes in nickel-exposed hTLR-4-positive female mice, it was also determined that a greater number of these cells expressed an activated phenotype at 10 d. In VC females, only 2.2% of the CD8^+^ T-cell population expressed a CD44^hi^ activated phenotype, whereas 16.1% of the population became activated following nickel exposure. Interestingly, despite the lack of significant change in lymph node B-cell proportionality at 10 d, female hTLR-4-positive mice exposed to nickel were observed to have a significant increase in percentage of B-cells bearing a CD86^hi^ activated phenotype at the same timepoint. NiSO_4_ exposure resulted in 22.1% activation of lymph node B-cells at 10 d, whereas only 4.1% of the same cell population was activated in same-sex VC animals.

Further, although no significant alterations in lymphocyte subset proportionality were observed in the nodes of nickel-exposed hTLR-4-positive male mice at 10 d, an increase in the proportion of activated CD8^+^ T-cells was seen. Compared to 3.1% of the lymph node CD8^+^ T-cell sub-population in VC mice, 8.8% of this cellular subset became activated in male NiSO_4_-exposed mice at 10 d.

### Spleen cell analysis

The spleen was collected from each animal at 10 d, weighed, processed, and subjected to phenotypic analysis by flow cytometry using the same technique as that used to evaluate lymph node cells. Results from analysis of the spleen are shown in Table 2. No significant alterations in spleen weight were seen at 10 d, irrespective of sex or treatment. Similar to observations within the lymph nodes, only hTLR-4-positive females exposed to NiSO_4_ exhibited significant alterations in the profile of lymphocyte subsets within the spleen at 10 d. Compared to VC animals, NiSO_4_-exposed females exhibited a significantly greater proportion of B-cells (61.1 vs. 52.3%) and a significantly decreased proportion of “other” cell types (6.0 vs. 12.2%) comprising the spleen at Day 10.

The only notable alterations in lymphocyte activation status observed in the spleens of hTLR-4-positive mice at 10 d involved splenic B-cell populations. In females, the significant increase in B-cell proportionality was accompanied by an increase in the number of cells expressing a CD86^hi^ phenotype. In VC mice, only 5.1% of B-cells in the spleen expressed elevated levels of the activation marker, whereas 30.5% of the B-cells became activated in NiSO_4_-exposed animals. A similar trend was observed in male mice, though the response was not as pronounced as noted in female mice. The percentage of activated B-cells in male hTLR-4-positive mice exposed to NiSO_4_ reached 16.6% by 10 d, compared to 5.3% in same-sex VC animals.

### Blood and serum analysis

Results from the phenotypic differentiation of circulating leukocyte populations at 10 d is shown in Table 3. Although total leukocyte number was increased in both female and male mice exposed to NiSO_4_ when compared to same-sex VC control values, the increase was only significant in female mice (6.17 × 10^3^/μl vs. 3.45 × 10^3^/μl blood). Similarly, the only significant alterations in leukocyte subset proportionality were noted in female hTLR-4-positive mice. NiSO_4_ exposure was associated with an increase in the number [and percentage] of circulating lymphocytes (5.24 × 10^3^/μl [84.8%] vs. 2.59 × 10^3^/μl [74.8%]), as well as a significant decrease in the number and percentage of circulating neutrophils (0.56 × 10^3^/μl [9.0%] vs. 0.67 × 10^3^/μl [19.7%]).

**Figure 4.**
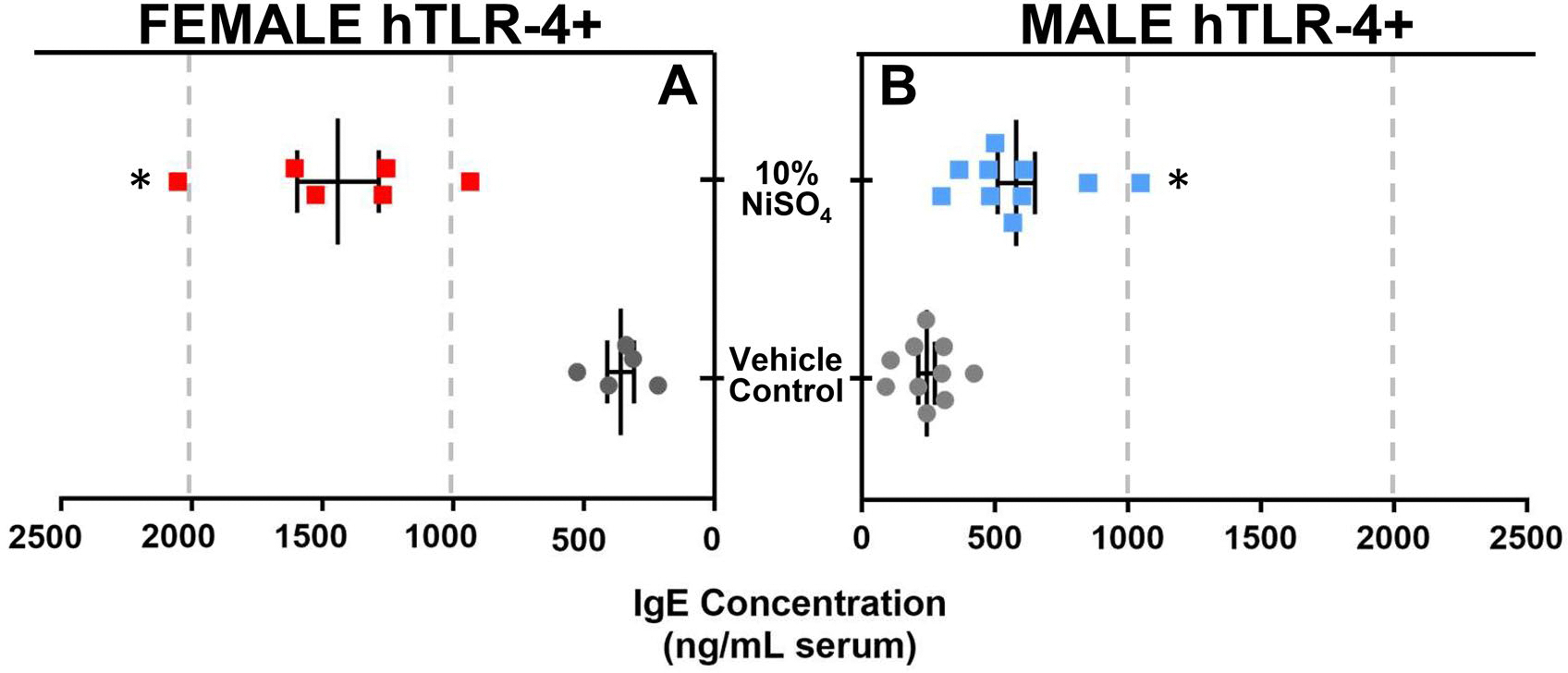
Total IgE levels in female (A) and male (B) hTLR-4-positive mice at the 10-d timepoint. In left panel (a), circulating IgE concentration (ng/ml serum) is shown for female mice exposed to vehicle (gray dots) or 10% NiSO_4_ (red squares). In right panel (B), concentrations are shown for male mice exposed to vehicle (gray dots) or 10% NiSO_4_ (blue squares). *n* = 6–10, *p* < 0.05, *statistically different from same-sex control group.

The concentration of total circulating IgE was also assessed at 10 d in serum collected from each animal (Figure 4). Both female and male hTLR-4-positive mice exposed to NiSO_4_ exhibited signifi-cantly elevated IgE values at 10 d; however, female mice, again, exhibited a greater maximal response over males. Average IgE concentration was determined to be 1442 ng/ml serum in NiSO_4_-exposed females, compared to 359 ng/ml in the VC group. In males, NiSO_4_ exposure led to an average of 580 ng/ml serum, compared to 243 ng/ml in same-sex VC controls at 10 d.

**Figure 5.**
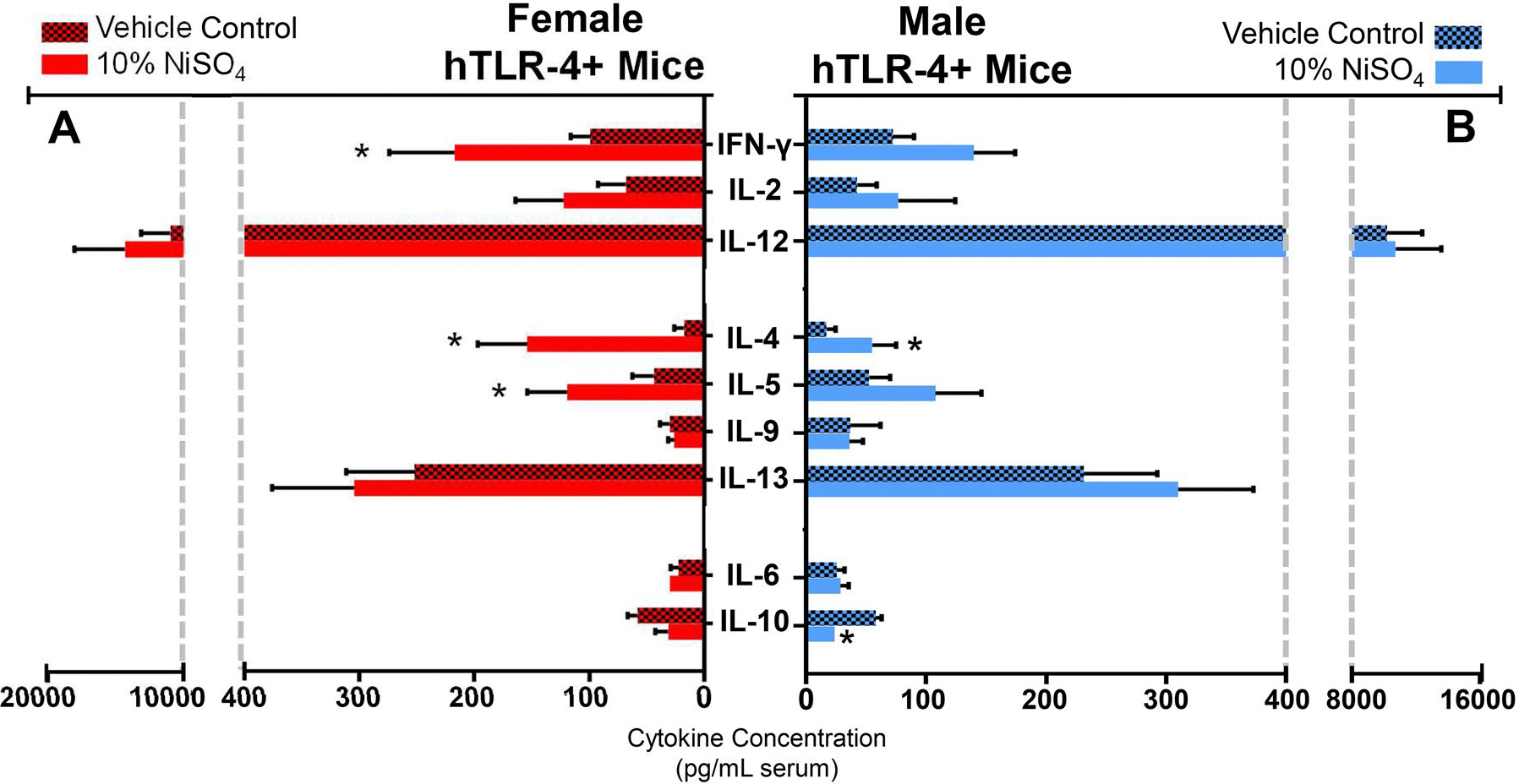
Serum cytokine concentrations in female (A) and male (B) hTLR-4-positive mice at the 10-d timepoint. Cytokines quantified include prototypical T_H_1 (IFNg, IL-2, IL-12) and T_H_2 (IL-4, IL-5, IL-9, IL-13) cytokines, as well as two miscellaneous cytokines (IL-6, IL-10). In left panel (a), cytokine concen-trations (pg/ml serum) are shown for female mice exposed to vehicle (hatched red bars) or 10% NiSO_4_ (solid red bars). In right panel (B), concentrations are shown for male mice exposed to vehicle (hatched blue bars) or 10% NiSO_4_ (solid blue bars). *n* = 6–10, *p* < 0.05, *statistically different from same-sex control group.

**Figure 6.**
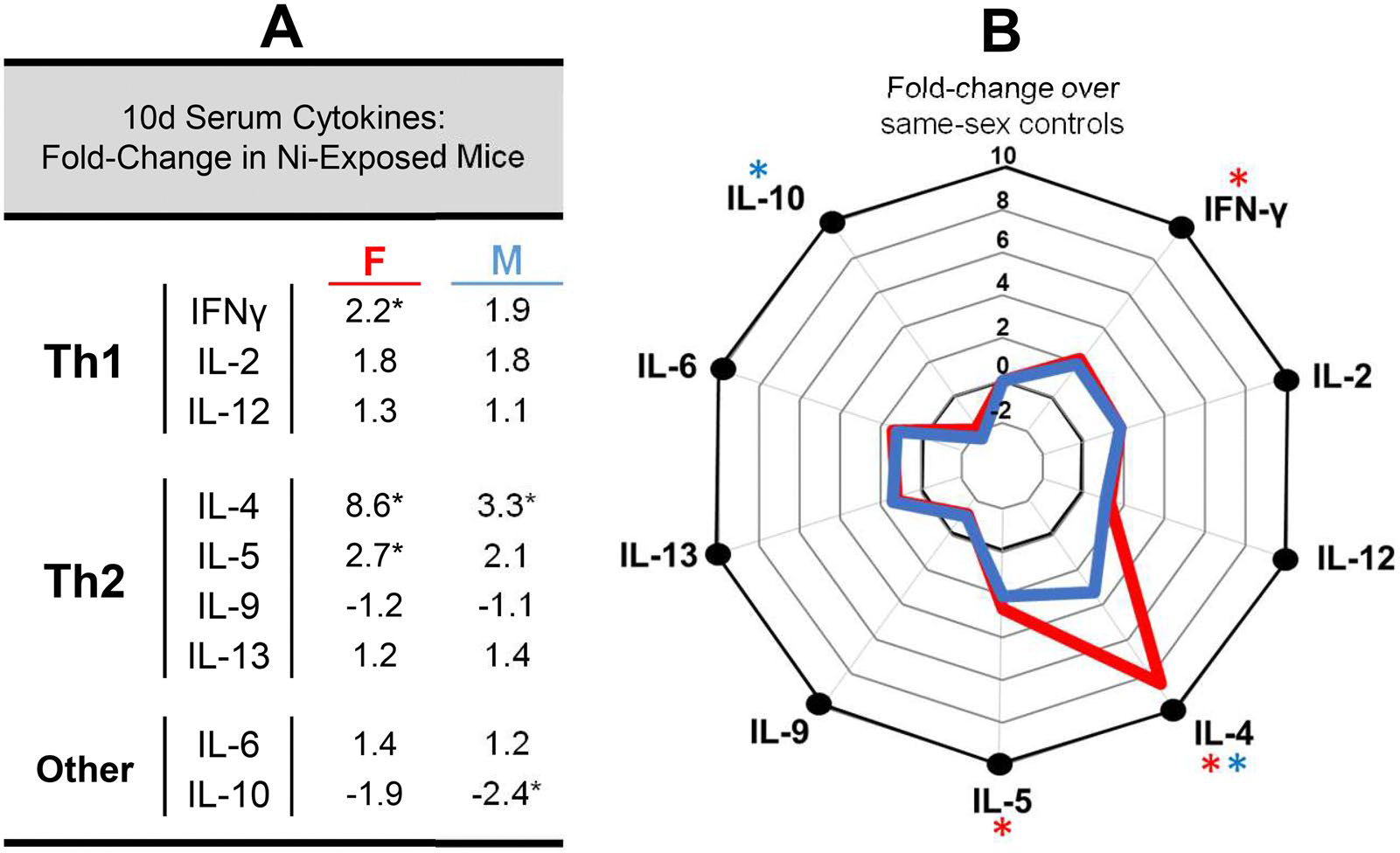
Nickel-induced alterations in serum cytokine profiles of hTLR-4-positive mice at the 10-d timepoint. Changes in circulating cytokine concentrations (expressed as fold-change over same-sex vehicle control animals) are listed in (a) for NiSO_4_-exposed mice of both sexes. A radar plot illustrating these changes in female (red line) and male (blue line) animals is shown in B. *n* = 6–10, *p* < 0.05, *statistically different from same-sex control group.

Finally, concentrations of circulating cytokines were evaluated using serum from each animal to better assess the response to nickel sensitization at 10 d. Levels of several prototypical T_H_1 (IFNγ, IL-2, IL-12) and T_H_2 (IL-4, IL-5, IL-9, IL-13) cytokines were evaluated, along with concentrations of two pleiotropic (i.e., IL-6, IL-10) cytokines with both pro- and anti-inflammatory potential. Serum cytokine concentrations are shown in Figure 5 (raw values) and Figure 6 (fold-increase over control).

Several sex-dependent alterations in the serum cytokine profile following nickel exposure were evident at 10 d (Figures 5 and 6). Both female and male hTLR-4-positive mice exposed to NiSO_4_ exhibited a significant increase in circulating IL-4 levels compared to same-sex control values. This response was more pronounced in female (8.6-fold increase) compared to in male mice (3.3-fold increase). In female mice, nickel exposure also led to significant increases in IFNγ (2.2-fold increase) and IL-5 (2.7-fold increase) levels. In male mice, the only other significant change was a decrease in IL-10 levels (2.4-fold decrease).

## Discussion

Overall, results from the LLNA indicate that expression of hTLR-4 by mice confers an elevated degree of immunological responsivity to nickel compared to responses seen in non-carrier littermates and wild-type mice used in previous studies (Kimber et al. 1990; Ikarashi, Ohno et al. 1992; Ikarashi, Tsuchiya et al. 1992; Ikarashi et al. 1993; Mandervelt et al. 1997; Basketter et al. 1999). In female mice, hTLR-4 expression was associated with a 7.4-fold increase in lymph node proliferation following exposure to 10% NiSO_4_ (SI of 0.5 in hTLR-4-negative mice, SI of 3.7 in hTLR-4-positive mice). Similarly, in male mice, hTLR-4 expression was associated with a 4.8-fold increase in lymphocyte proliferation (SI of 0.8 in hTLR-4-negative mice, SI of 3.8 in hTLR-4-positive mice) following exposure to 10% NiSO_4_. Though these values indicate that hTLR-4-positive mice were successfully sensitized to the metal (SI ≥ 3), the maximum observed SI of 3.8 is considered a modest response in the LLNA. Based on these results, NiSO_4_ would be classified as a mild sensitizer.

At present, there is very little information available regarding the allergenic potency of nickel salts during the sensitization phase of ACD in humans; given the prevalence of nickel allergy (afflicting nearly 20% of the global population), this knowledge gap is somewhat surprising. It has been suggested though, that the prevalence of nickel allergy in the general population may be more reflective of the frequency at which humans come into contact with nickel and the nature of these interactions, than its inherent immunogenicity (Basketter 2021). This theory is supported by the well-established correlation between nickel allergy and ear/body piercings and the higher frequency of nickel allergy in women over men (Ehrlich et al. 2001). Several of the major metal alloys used to make jewelry contain nickel in various quantities, and introduction of these objects into a piercing site can promote sensitization as a result of: (1) continuous, low-level release of haptenic ions from the object, (2) increased potential for ion absorption due to compromised skin barrier integrity (or direct access to circulation in some cases), (3) localized inflammation and irritation from piercing trauma (e.g., increased APC, elevated DAMP/alarmin presence) that can amplify immune responsivity, and (4) the extended duration of contact typically associated with wearing jewelry, which can range from hours/day to the entire day. Since the LLNA cannot account for these exposure dynamics seen in humans, it is important to note that the SI values obtained from this study only constitute a single component of the allergenic profile associated with nickel salts.

Similar to findings from the LLNA study, a greater degree of immunological responsivity was observed in female mice compared to males in the 10-d study. For example, both female and male hTLR-4-positive mice exhibited significantly elevated levels of circulating total IgE following skin exposure to 10% NiSO_4_, but IgE responses were more pronounced in female mice compared to male mice. Although ACD is a delayed-type, cell-mediated hypersensitivity response that would not be expected to elicit any notable alterations in IgE production, similar findings have been reported previously (Arts et al. 1997). At present, there are multiple different assays that have been validated for use to assess compounds with suspected skin-sensitizing potential (e.g., LLNA, GPMT, Buehler, KeratinoSens, h-CLAT) (Vandebriel and van Loveren 2010). Comparatively, there has yet to be a single assay validated for the selective identification of potential respiratory sensitizers. Although many approaches have been proposed, none have proven effective enough to be endorsed by major regulatory agencies or standards organizations like the OECD (Chary et al. 2018). As a result, many of the assays currently used by researchers to investigate potential asthmagens resemble those traditionally used to study skin sensitizers, but with slight modifications. For example, it has been shown that by incorporating additional endpoints into the LLNA (e.g., serum IgE, circulating cytokine levels), these markers can help identify compounds that may pose a particular risk for the development of antibody-mediated respiratory allergy (Dearman et al. 2003). Trimellitic anhydride is a dermal and respiratory sensitizer that has been shown to induce IgE production in rats following dermal exposure, while other compounds known to exclusively induce dermal allergy and irritant responses (e.g., HCA) fail to produce such antibody responses (Arts et al. 1997). Similarly, in mice, repeated dermal exposure to platinum salts (known to cause IgE-mediated asthma in workers) induces subsequent increases in serum IgE (Dearman et al. 1998). In accordance with this knowledge, the observation that NiSO_4_ exposure caused elevated circulating IgE levels 10 d post-exposure in the current study indicates the metal may have a potential to induce respiratory sensitivity as well as contact allergy. This conclusion is supported by existing know-ledge that respiratory exposure to nickel in the workplace can lead to development of metal-specific asthma in susceptible workers (Novey et al. 1983; Nieboer et al. 1984).

It is also interesting to note that the aforementioned studies that reported elevations in IgE following the incorporation of potential respiratory sensitizers into the LLNA were performed using T_H_2-dominant strains (Brown Norway rats, BALB/c mice) to provide an additional level of sensitivity to the assay. In this study, nickel exposure was associated with significant IgE increases despite use of the T_H_1-prone C57BL/6 strain. Since this strain is expected to prefer-entially mount T_H_1-dominant/cell-mediated immune responses that negatively regulate develop-ment of T_H_2/IgE-mediated reactions, the significant increase in IgE production detected in this study suggests that nickel may possess a greater potential for inducing asthmatic responses than previously thought; however, it should be noted that the specificity of IgE molecules was not evaluated in this study, so it remains unclear if the serum IgE elevations seen in the 10-d study are reflective of actual nickel-specific antibody production (de Mello et al. 2009).

It has been similarly suggested that, like IgE, serum cytokine responses following skin exposure to potential sensitizers may represent another useful endpoint for identifying substances with the capacity to cause ACD and or asthma (Dearman et al. 2003). Elevated production of Type 1 cytokines (e.g., IFNγ), for example, has been correlated with development of cell-mediated allergic skin responses like ACD (Dearman et al. 1996). Contrarily, increased production of Type 2 cytokines (e.g., IL-4, IL-10) appear to be reflective of the potential for respiratory sensitization and IgE-mediated asthmatic responses (Vandebriel et al. 2000; Van Och et al. 2002). The mixed-type cytokine responses observed at 10 d in hTLR-4-positive mice exposed to 10% NiSO_4_ suggest that this compound may have the capacity to cause both ACD and asthma. This finding is consistent with existing knowledge about the immune-toxic effects of nickel, as it is one of the most common inducers of ACD in humans and is also known to cause asthmatic responses, particularly in workers exposed to the metal in the form of airborne particulates and fumes in occupational settings (Malo et al. 1982; Hong et al. 1986; Bright et al. 1997; Brera and Nicolini 2005). Further, the increase in circulating Type 2 cytokine concentra-tions seen at 10 d implies that skin exposure to soluble nickel salts may also prime the immune system for development of asthmatic responses upon subsequent encounters.

Similar to trends in other immune parameters evaluated in this study, the serum cytokine profiles characterized at 10 d also exhibited several notable sex-dependent discrepancies. Although male mice exhibited significantly increased circulating IL-4 levels at 10 d (3.3× higher than in VC-exposed males), a significant decrease in IL-10 levels was observed simultaneously. Compared to male mice, the increase in IL-4 observed in female mice at 10 d was far more pronounced (8.6x higher than in VC controls). This increase was accompanied by significant elevations in IL-5, another key Type 2 cytokine involved in IgE-mediated allergic responses. Interestingly, concurrent to increased production of these T_H_2 cytokines, a significant increase in serum IFNγ was also observed in these animals. These observations further emphasize a critical role for sex in shaping the nature and magnitude of immune responses that develop following nickel exposure, which should be considered in future studies.

Although the results of this study support a critical role for TLR-4 structure in skin sensitization by nickel, many other factors contribute to this process as well. This investigation served as a proof-of-concept study with the hTLR-4 mouse model for future examinations of the more precise immunological mechanisms involved in nickel allergy, and thus, has many shortcomings. The findings of this study have helped identify additional biomarkers of interest that will direct future studies and better elucidate how nickel induces hypersensitivity responses in some individuals. For example, a potential explanation for the increased responsivity to nickel by females may be reflective of sex-dependent differences in receptor density. Additional information can also be garnered from an expanded cytokine profile, *ex vivo* stimulation of lymphocytes, and antibody subset profiling (e.g., IgG_1_, IgG_2a_).

Overall, the results obtained from this study suggest that the novel hTLR-4 mouse model represents a viable approach for future studies of nickel allergy *in vivo*. Expression of humanized TLR-4 was associated with enhanced immunological responsivity to soluble forms of the metal in both sexes of mice, more closely emulating humans’ susceptibility to nickel allergy than observed in previous efforts. Moreover, the study results were also able to identify several unique mechanisms by which the immune response to nickel differs in a sex-dependent manner (Pilar Alcón et al. 1991; Macia and Hernández 1995; Vahter et al. 2007). Since both the nature and magnitude of immune reactions to nickel can vary significantly between female and male mice, sex constitutes an additional variable that must be considered when selecting the appropriate animal model for future investigations of nickel biological effects *in vivo.*

## Figures and Tables

**Table 1.** Auricular lymph node cell phenotypes in hTLR-4 positive mice at 10d.

	Female		Male	
	VC	NiSO_4_	VC	NiSO_4_

Total LN Cell #	3.32±0.40 × 10^6^	1.98±0.33 × 10^7^ *	2.99±0.51 × 10^6^	9.59±2.14 × 10^6^ *
CD4+ T-cells	51.2±2.5%	44.5±4.4%*	49.9±3.6%	47.2±5.1%
% act (CD44^hi^)	2.0±1.1%	5.1±2.0%	1.9±0.5%	3.3±1.4%
cD8+ T-cells	20.1±1.3%	25.1±2.0%*	22.7±3.2%	24.0±3.3%
% act (CD44^hi^)	2.2±1.1%	16.1±4.4%*	3.1±0.7%	8.8±2.2%*
B-cells	22.4±2.1%	26.3±3.4%	23.3±1.8%	25.4±3.5%
% act (CD86^hi^)	4.1±1.5%	22.1±4.5%*	3.3±0.9%	6.2±2.2%
NK cells	1.1±0.1 %	1.0±0.1%	0.9±0.2%	0.8±0.1%
Other cells	5.2±1.1%	3.1±0.4%	3.2±0.4%	2.6±0.5%

Phenotypic analysis of auricular lymph node cells from female and male hTLR-4 positive mice at the 10 day timepoint. For each group, total lymph node cell number is reported, along with the percentage of cD4+ T-cells, cD8+ T-cells, B-cells, NK cells, and other cell types comprising the nodes. In addition, the percentage of each lymphocyte subset bearing an activated phenotype (cD44^hi^ for T-cells and CD86^hi^ for B-cells) is reported. *n* = 6–10, *p* < 0.05,

* indicates statistically different from same-sex control group.

**Table 2.** Spleen cell phenotypes in hTLR-4 positive mice at 10d.

	Female		Male	
	VC	NiSO_4_	VC	NISO_4_

Spleen weight (g)	0.082±0.008	0.086±0.011	0.102±0.023	0.099±0.008
CD4+ T-cells	20.3±2.1%	17.4±1.4%	21.1±3.0%	18.4±1.9%
% act (cD44^hi^)	3.0±1.5%	5.1±2.0%	2.9±1.5%	4.0±1.6%
CD8+ T-cells	13.2±1.1%	14.0±2.0%	15.0±1.5%	14.7±2.1%
% act (cD44^hi^)	4.2±2.0%	7.1±3.4%	5.1±2.7%	3.8±2.1%
B-cells	52.3±3.4%	61.1±2.4%*	50.0±4.1%	54.2±2.2%
% act (cD86^hi^)	5.1±2.5%	30.5±8.6%*	5.3±1.9%	16.6±5.2%*
NK cells	2.0±0.2%	1.5±0.4%	2.2±0.2%	1.8±0.3%
Other cells	12.2±3.2%	6.0±1.1%*	11.7±2.5%	10.9±1.1%

Phenotypic analysis of spleen cells from female and male hTLR-4 positive mice at the 10 day timepoint. For each group, average spleen weight is reported, along with the percentage of cD4+ T-cells, cD8+ T-cells, B-cells, NK cells, and other cell types comprising the spleen. In addition, the percentage of each lymphocyte subset bearing an activated phenotype (cD44^hi^ for T-cells and cD86^hi^ for B-cells) is reported. *n* = 6–10, *p* < 0.05,

* indicates statistically different from same-sex control group.

**Table 3.** Circulating leukocyte populations in hTLR-4 positive mice at 10d.

Sex	Treatment group	Absolute number (k/μL blood)					Total leukocyte number	Percentage of total leukocytes				
		Lymph	Neutr	Mono	Eos	Baso		Lymph	Neutr	Mono	Eos	Baso

Female	VC	2.59 ± 0.51	0.67 ± 0.04	0.11 ± 0.01	0.07 ± 0.01	0.00 ± 0.00	3.45 ± 0.24	74.8% ± 3.3%	19.7% ± 2.6%	3.3% ± 0.5%	2.1% ± 0.3%	0.1% ± 0.0%
	10% NiSO_4_	5.24* ± 0.42	0.56* ± 0.03	0.21 ± 0.02	0.16 ± 0.02	0.01 ± 0.00	6.17 ± 0.42*	84.8%* ± 4.1%	9.0%* ± 1.1%	3.4% ± 0.3%	2.7% ± 0.5%	0.1% ± 0.0%
Male	VC	2.89 ± 0.65	1.05 ± 0.02	0.36 ± 0.02	0.07 ± 0.01	0.01 ± 0.01	4.37 ± 0.50	65.9% ± 3.0%	24.5% ± 2.2%	7.9% ± 1.1%	1.6% ± 0.2%	0.2% ± 0.0%
	10% NiSO_4_	3.77 ± 0.34	1.04 ± 0.03	0.43 ± 0.02	0.05 ± 0.01	0.01 ± 0.00	5.29 ± 0.48	70.6% ± 2.7%	20.4% ± 1.3%	8.0% ± 0.9%	0.8% ± 0.1%	0.1% ± 0.0%

Phenotypic analysis of circulating leukocyte populations in female and male hTLR-4 positive mice at the 10 day timepoint. Absolute number of lymphocytes, neutrophils, monocytes, eosinophils, and basophils was determined for each animal from whole blood. total leukocyte number and percentages of each cell subset where then calculated and averaged within each group. *n* = 6–10, *p* < 0.05,

* indicates statistically different from same-sex control group.

## Abbreviations:

ACD: allergic contact dermatitis
ANOVA: analysis of variance
APC: antigen-presenting cell
DAMP: damage-associated molecular pattern
DC: dendritic cell
DMSO: dimethyl sulfoxide
DNFB: dinitrofluorobenzene
DPM: disintegrations per minute
GPMT: guinea pig maximization test
HCA: hexylcinnamaldehyde
HMW: high molecular weight
hTLR-4: humanized Toll-like receptor 4
IFN: interferon
IgE: immunoglobulin E
IL: interleukin
LLNA: Local Lymph Node Assay
LPS: lipopolysaccharide
MCP-1: monocyte chemotactic protein-1
NF-κB: nuclear factor kappa B
NIOSH: National Institute for Occupational Safety and Health
NK: natural killer cell
OECD: Organization for Economic Co-operation and Development
PAMP: pathogen-associated molecular pattern
PRR: pathogen recognition receptor
ROS: reactive oxygen species
SI: stimulation index
TCA: trichloroacetic acid
TDI: toluene diisocyanate
T_H_: T-helper cell
TLR-4: Toll-like receptor-4
VC: vehicle control

## References

[R1] Abeck D, Traenckner I, Steinkraus V, Vieluf D, Ring J. 1993. Chronic urticaria due to nickel intake. Acta Derm Venereol. 73(6):438–439. doi: 10.2340/0001555573438439. 7906457

[R2] Ahlström MG, Thyssen JP, Wennervaldt M, Menné T, Johansen JD. 2019. Nickel allergy and allergic contact dermatitis: A clinical review of immunology, epidemiology, exposure, and treatment. Contact Dermatitis. 81(4):227–241. doi: 10.1111/cod.13327. 31140194

[R3] Alcón MP, Arola L, Mas A. 1991. Response to acute nickel toxicity in rats as a function of sex. Biol Met. 4(3):136–140. doi: 10.1007/BF01141303. 1931431

[R4] Almpanis G, Tsigkas G, Koutsojannis C, Mazarakis A, Kounis G, Kounis N. 2010. Nickel allergy, Kounis syndrome, and intracardiac metal devices. Int J Cardiol. 145(2):364–365. doi: 10.1016/j.ijcard.2010.02.038. 20207036

[R5] Anderson S, Long C, Dotson G. 2017. Occupational allergy. EMJ. 2:65–71. doi: 10.33590/emj/10311285. 30976662 PMC6454566

[R6] Anderson S, Wells J, Fedorowicz A, Butterworth L, Meade B, Munson A. 2007. Evaluation of the contact and respiratory sensitization potential of volatile organic compounds generated by simulated indoor air chemistry. Toxicol Sci. 97(2):355–363. doi: 10.1093/toxsci/kfm043. 17347135

[R7] Arts J, Dröge S, Spanhaak S, Bloksma N, Penninks A, Kuper C. 1997. Local lymph node activation and IgE responses in Brown Norway and Wistar rats after dermal application of sensitizing and non-sensitizing chemicals. Toxicology. 117(2–3):229–234. doi: 10.1016/s0300-483x(96)03576-7. 9057902

[R8] Asher M, Montefort S, Björkstén B, Lai C, Strachan D, Weiland S, Williams H. 2006. Worldwide time trends in the prevalence of symptoms of asthma, allergic rhinoconjunctivitis, and eczema in childhood: ISAAC Phases One and Three repeat multi-country cross-sectional surveys. Lancet. 368(9537):733–743. doi: 10.1016/S0140-6736(06)69283-0. 16935684

[R9] Basketter D 2021. Nickel: Intrinsic skin sensitization potency and relation to prevalence of contact allergy. Dermatitis. 32(2):71–77. doi: 10.1097/DER.0000000000000666. 32826408 PMC7982139

[R10] Basketter D, Lea L, Cooper K, Ryan C, Gerberick G, Dearman R, Kimber I. 1999. Identification of metal allergens in the local lymph node assay. Am J Contact Dermatitis. 10(4):207–212. doi: 10.1097/01206501-199912000-00005. 10594296

[R11] Brera S, Nicolini A. 2005. Respiratory manifestations due to nickel. Acta Otorhinolaryn Ital. 25(2):113–115. https://old.actaitalica.it/issues/2005/2-05/Brera.pdf. PMC2639879 16116834

[R12] Bright P, Burge P, O’Hickey S, Gannon P, Robertson A, Boran A. 1997. Occupational asthma due to chrome and nickel electroplating. Thorax. 52(1):28–32. doi: 10.1136/thx.52.1.28. 9039236 PMC1758409

[R13] Buyukozturk S, Gelincik A, Demirtürk M, Erdoğdu D, Pur L, Colakoğlu B, Deniz G, Erdem Kuruca S. 2013. Nickel dental alloys can induce laryngeal edema attacks: a case report. J Dermatol. 40(9):740–742. doi: 10.1111/1346-8138.12210. 23834453

[R14] Candura S, Locatelli C, Butera R, Gatti A, Fasola D, Manzo L. 2001. Widespread nickel dermatitis from inhalation. Contact Dermatitis. 45(3):174–175. doi: 10.1034/j.1600-0536.2001.045003174.x. 11553151

[R15] Chary A, Hennen J, Klein S, Serchi T, Gutleb A, Blömeke B. 2018. Respiratory sensitization: toxico-logical point of view on the available assays. Arch Toxicol. 92(2):803–822. doi: 10.1007/s00204-017-2088-5. 29038838

[R16] Cochrane S, Arts J, Ehnes C, Hindle S, Hollnagel H, Poole A. Suto H, Kimber I. 2015. Thresholds in chemical respiratory sensitisation. Toxicology. 333:179–194. https://www.sciencedirect.com/science/article/pii/S0300483X15000815?via%3Dihub 25963507 10.1016/j.tox.2015.04.010

[R17] Dalal V, Chhimwal R, Verma R, Suresh B. 2014. Evaluation of sex sensitivity in local lymph node assay using acephate and α-hexylcinnamaldehyde. IOSRJESTFT. 8(6):31–37. doi: 10.9790/2402-08633137.

[R18] de Mello L, Bechara M, Solé D, Rodrigues V. 2009. T_H_1/T_H_2 balance in concomitant immediate and delayed-type hypersensitivity diseases. Immunol Lett. 124(2):88–94. doi: 10.1016/j.imlet.2009.04.011. 19433108

[R19] Dearman R, Basketter D, Kimber I. 1996. Characterization of chemical allergens as a function of divergent cytokine secretion profiles induced in mice. Toxicol Appl Pharmacol. 138(2):308–316. doi: 10.1006/taap.1996.0129. 8658532

[R20] Dearman R, Basketter D, Kimber I. 1998. Selective induction of Type 2 cytokines following topical exposure of mice to platinum salts. Food Chem Toxicol. 36(3):199–207. doi: 10.1016/s0278-6915(97)00143-9. 9609393

[R21] Dearman R, Skinner R, Humphreys N, Kimber I. 2003. Methods for the identification of chemical respiratory allergens in rodents: comparisons of cytokine profiling with induced changes in serum IgE. J Appl Toxicol. 23(4):199–207. doi: 10.1002/jat.907. 12884401

[R22] Dierick B, van der Molen T, Flokstra-de Blok B, Muraro A, Postma M, Kocks J, van Boven J. 2020. Burden and socioeconomics of asthma, allergic rhinitis, atopic dermatitis and food allergy. Expert Rev Pharmacoecon Outcomes Res. 20(5):437–453. doi: 10.1080/14737167.2020.1819793. 32902346

[R23] Ditrichova D, Kapralova S, Tichy M, Ticha V, Dobesova J, Justova E, Eber M, Pirek P. 2007. Oral lichenoid lesions and allergy to dental materials. Biomed Pap Med Fac Univ Palacky Olomouc Czech Repub. 151(2):333–339. doi: 10.5507/bp.2007.057. 18345274

[R24] Eguchi T, Kumagai K, Kobayashi H, Shigematsu H, Kitaura K, Suzuki S, Horikawa T, Hamada Y, Ogasawara K, Suzuki R. 2013. Accumulation of invariant NKT cells into inflamed skin in a novel murine model of nickel allergy. Cell Immunol. 284(1–2):163–171. doi: 10.1016/j.cellimm.2013.07.010. 23978680

[R25] Ehrlich A, Kucenic M, Belsito D. 2001. Role of body piercing in the induction of metal allergies. Am J Contact Dermatitis. 2001(12):151–155. 10.1053/ajcd.2001.22774 11526520

[R26] Estlander T, Kanerva L, Tupasela O, Keskinen H, Jolanki R. 1993. Immediate and delayed allergy to nickel with contact urticaria, rhinitis, asthma, and contact dermatitis. Clin Exp Allergy. 23(4):306–310. doi: 10.1111/j.1365-2222.1993.tb00327.x. 8319128

[R27] Franzen D, Lang C, Agorastos N, Freitag L, Kohler M, Schmid-Grendelmeier P. 2017. Evaluation of nickel release from endobronchial valves as a possible cause of hypersensitivity pneumonitis in a patient treated with bronchoscopic lung volume reduction. Int Arch Allergy Immunol. 174(3–4):144–150. doi: 10.1159/000481986. 29136621

[R28] Hajjar AM, Ernst RK, Fortuno ES, Brasfield AS, Yam CS, Newlon LA, Kollmann TR, Miller SI, Wilson CB. 2012. Humanized TLR4/MD-2 Mice reveal LPS recognition differentially impacts susceptibility to *Yersinia pestis* and *Salmonella enterica*. PLoS Pathog. 8(10):e1002963. doi: 10.1371/journal.ppat.1002963. 23071439 PMC3469661

[R29] Hong C, Oh S, Lee H, Huh K, Lee S. 1986. Occupational asthma caused by nickel and zinc. Korean J Intern Med. 1(2):259–262. doi: 10.3904/kjim.1986.1.2.259. 3154623 PMC4536724

[R30] Ikarashi Y, Ohno K, Tsuchiya T, Nakamura A. 1992. Differences of draining lymph node cell proliferation among mice, rats and guinea pigs following exposure to metal allergens. Toxicology. 76(3):283–292. doi: 10.1016/0300-483X(92)90196-L. 1471161

[R31] Ikarashi Y, Tsuchiya T, Nakamura A. 1992. Detection of contact sensitivity of metal salts using the murine local lymph node assay. Toxicol Lett. 62(1):53–61. doi: 10.1016/0378-4274(92)90078-x. 1509507

[R32] Ikarashi Y, Tsukamoto Y, Tsuchiya T, Nakamura A. 1993. Influence of irritants on lymph node cell proliferation and the detection of contact sensitivity to metal salts in the murine local lymph node assay. Contact Dermatitis. 29(3):128–132. doi: 10.1111/j.1600-0536.1993.tb03509.x. 8222623

[R33] Jacob S, Goldenberg A, Pelletier J, Fonacier L, Usatine R, Silverberg N. 2015. Nickel allergy and our children’s health: A review of indexed cases and a view of future prevention. Pediatr Dermatol. 32(6):779–785. doi: 10.1111/pde.12639. 26212605

[R34] Johansen P, Wäckerle-Men Y, Senti G, Kündig T. 2010. Nickel sensitisation in mice: A critical appraisal. J Dermatol Sci. 58(3):186–192. doi: 10.1016/j.jdermsci.2010.03.011. 20456924

[R35] Kimber I, Basketter D, McFadden J, Dearman R. 2011. Characterization of skin sensitizing chemicals: a lesson learnt from nickel allergy. J Immunotoxicol. 8(1):1–2. doi: 10.3109/1547691X.2010.531298. 21067469

[R36] Kimber I, Bentley A, Hilton J. 1990. Contact sensitization of mice to nickel sulphate and potassium dichromate. Contact Dermatitis. 23(5):325–330. doi: 10.1111/j.1600-0536.1990.tb05166.x. 2096022

[R37] Kusaka Y, Nakano Y, Shirakawa T, Fujimura N, Kato M, Heki S. 1991. Lymphocyte transformation test with nickel in hard metal asthma: Another sensitizing component of hard metal. Ind Health. 29(4):153–160. doi: 10.2486/indhealth.29.153. 1814868

[R38] Lamb C, Ratner P, Johnson C, Ambegaonkar AJ, Joshi AV, Day D, Sampson N, Eng B. 2006. Economic impact of workplace productivity losses due to allergic rhinitis compared with select medical conditions in the United States from an employer perspective. Curr Med Res Opin. 22(6):1203–1210. doi: 10.1185/030079906X112552. 16846553

[R39] Malo J, Cartier A, Doepner M, Nieboer E, Evans S, Dolovich J. 1982. Occupational asthma caused by nickel sulfate. J Allergy Clin Immunol. 69(1 Pt 1):55–59. doi: 10.1016/0091-6749(82)90088-4. 7054253

[R40] Malo J, Cartier A, Gagnon G, Evans S, Dolovich J. 1985. Isolated late asthmatic reaction due to nickel sulphate without antibodies to nickel. Clin Allergy. 15(2):95–99. doi: 10.1111/j.1365-2222.1985.tb02261.x. 3995728

[R41] Mandervelt C, Clottens F, Demedts M, Nemery B. 1997. Assessment of the sensitization potential of five metal salts in the murine local lymph node assay. Toxicology. 120(1):65–73. doi: 10.1016/S0300-483X(97)03629-9. 9160110

[R42] Moon J, Reeder M, Atwater A. 2021. Contact allergy to nickel: still #1 after all these years. Cutis. 107(1):12–15. doi: 10.12788/cutis.0156. 33651861

[R43] Nieboer E, Evans S, Dolovich J. 1984. Occupational asthma from nickel sensitivity: II. Factors influencing the interaction of Ni^2+^, HSA, and serum antibodies with nickel-related specificity. Br J Ind Med. 41(1):56–63. doi: 10.1136/oem.41.1.56. 6691936 PMC1009236

[R44] Novey H, Habib M, Wells I. 1983. Asthma and IgE antibodies induced by chromium and nickel salts. J Allergy Clin Immunol. 72(4):407–412. doi: 10.1016/0091-6749(83)90507-9. 6413567

[R45] Nucera E, Chini R, Rizzi A, Schiavino D, Buonomo A, Aruanno A, Ricci R, Mangiola F, Campanale M, Gasbarrinin A, 2019. Eosinophilic oesophagitis (in nickel-allergic patient) regressed after nickel oral desensitization: a case report. Intl J Immunopathol Pharmacol. 33:205873841982. 10.1177/2058738419827771 PMC6407324 30834798

[R46] Pawankar R, Canonica G, Holgate S, Lockey R. 2013. WAO White Book on Allergy 2011–2012: executive Summary. Milwaukee, WI: World Allergy Organization.

[R47] Pföhler C, Vogt T, Müller C. 2016. Contact allergic gastritis: Rare manifestation of a metal allergy. Hautarzt. 67(5):359–364. doi: 10.1007/s00105-016-3773-7. 26909810

[R48] Prescott S, Pawankar R, Allen K, Campbell D, Sinn J, Fiocchi A, Ebisawa M, Sampson H, Beyer K, Lee B. 2013. Global survey of changing patterns of food allergy burden in children. World Allergy Organ J. 6(1):21. doi: 10.1186/1939-4551-6-21. 24304599 PMC3879010

[R49] Roach K, Roberts J. 2022. A comprehensive summary of disease variants implicated in metal allergy. J Toxicol Environ Health B Crit Rev. 25(6):279–341. doi: 10.1080/10937404.2022.2104981. 35975293 PMC9968405

[R50] Sánchez-Borges M, Martin B, Muraro A, Wood R, Agache I, Ansotegui I, Casale T, Fleisher T, Hellings P, Papadopoulos N, 2018. The importance of allergic disease in public health: An iCAALL statement. World Allergy Organ J. 11(1):8. doi: 10.1186/s40413-018-0187-2. 29743965 PMC5921992

[R51] Schmidt M, Martin S, Freudenberg M, Goebeler M. 2011. Animal models for nickel allergy. Nature Nanotech. 6(9):533–533. doi: 10.1038/nnano.2011.143. 21897378

[R52] Schmidt M, Raghavan B, Müller V, Vogl T, Fejer G, Tchaptchet S, Keck S, Kalis C, Nielsen PJ, Galanos C, 2010. Crucial role for human Toll-like receptor 4 in the development of contact allergy to nickel. Nat Immunol. 11(9):814–819. doi: 10.1038/ni.1919. 20711192

[R53] Serebrisky D, Wiznia A. 2019. Pediatric asthma: A global epidemic. Ann Global Health. 85:6. 10.5334/aogh.2416 PMC7052318 30741507

[R54] Tramontana M, Bianchi L, Hansel K, Agostinelli D, Stingeni L. 2020. Nickel allergy: epidemiology, patho-mechanism, clinical patterns, treatment and prevention programs. Endocr Metab Immune Disord Drug Targets. 20(7):992–1002. doi: 10.2174/1871530320666200128141900. 31994473

[R55] Vahter M, Akesson A, Lidén C, Ceccatelli S, Berglund M. 2007. Gender differences in the disposition and toxicity of metals. Environ Res. 104(1):85–95. doi: 10.1016/j.envres.2006.08.003. 16996054

[R56] van Och F, van Loveren H, de Jong W, Vandebriel R. 2002. Cytokine production induced by low-molecular-weight chemicals as a function of the stimulation index in a modified local lymph node assay: An approach to discriminate contact sensitizers from respiratory sensitizers. Toxicol Appl Pharmacol. 184(1):46–56. doi: 10.1006/taap.2002.9473. 12392968

[R57] Vandebriel RJ, De Jong WH, Spiekstra SW, Van Dijk M, Fluitman A, Garssen J, Van Loveren H. 2000. Assessment of preferential T_H_1 or T_H_2 induction by low molecular weight compounds using local lymph node assay in conjunction with RT-PCR and ELISA for IFNγ and IL-4. Toxicol Appl Pharmacol. 162(2):77–85. doi: 10.1006/taap.1999.8841. 10637130

[R58] Vandebriel R, van Loveren H. 2010. Non-animal sensitization testing: State-of-the-art. Crit Rev Toxicol. 40(5):389–404. doi: 10.3109/10408440903524262. 20180632

[R59] Verna N, Cavallucci E, Ramondo S, Paolini F, Grana M, Paganelli R, Di Gioacchino M. 2002. Asthma related to food ingested nickel. J Allergy Clin Immunol. 109(1):S235–S235. doi: 10.1016/S0091-6749(02)81849-8.

[R60] Warshaw E, Zhang A, DeKoven J, Maibach H, Belsito D, Sasseville D, Fowler J, Fransway A, Mathias T, Pratt M, 2019. Epidemiology of nickel sensitivity: Retrospective cross-sectional analysis of North American Contact Dermatitis Group data 1994–2014. J Am Acad Dermatol. 80(3):701–713. doi: 10.1016/j.jaad.2018.09.058. 30342160

[R61] Wöhrl S, Hemmer W, Focke M, Götz M, Jarisch R. 2003. Patch testing in children, adults, and the elderly: Influence of age and sex on sensitization patterns. Pediatr Dermatol. 20(2):119–123. doi: 10.1046/j.1525-1470.2003.20204.x. 12657006

[R62] Yaghoubi M, Adibi A, Safari A, FitzGerald J, Sadatsafavi M. 2019. The projected economic and health burden of uncontrolled asthma in the United States. Am J Respir Crit Care Med. 200(9):1102–1112. doi: 10.1164/rccm.201901-0016OC. 31166782 PMC6888652

[R63] Yoshiaki I, Toshie T, Akitada N. 1992. Detection of contact sensitivity of metal salts using the murine local lymph node assay. Toxicology letters. 62(1):53–61. 1509507 10.1016/0378-4274(92)90078-x

